# Supplementary material for: Are carbon emissions trading and green financial instruments synergistic? -Comprehensive quantitative research based on content analysis
Source: PLoS One. 2024 Mar 7;19(3):e0298601. doi: 10.1371/journal.pone.0298601 (PMC10919601; doi:10.1371/journal.pone.0298601)
Supplement: S1 Data — (ZIP) [file pone.0298601.s001.zip › Data/地级市Z_F工作报告.zip!/╣π╢1⁄2/╒╪╟∞╩╨/└┤╫╘╣1⁄2╓┌║┼í╛┬φ┐╦╩2╛▌═°í┐_2013.docx]

各位代表：

　　我代表肇庆市人民政府向大会作工作报告，请予审议，并请政协委员和其他列席人员提出意见。

　　2012年工作回顾

　　去年，面对复杂严峻的经济形势，在省委、省政府和市委的正确领导下，在市人大及其常委会和市政协的监督支持下，市政府深入贯彻落实科学发展观，围绕建设成为代表珠三角科学发展成果城市的奋斗目标，全面落实《珠三角规划纲要》，大力实施“两区引领两化”战略，全力保增长、促转型、保稳定、惠民生，推动经济社会持续平稳发展，基本完成市十二届人大一次会议确定的目标任务。

　　——全力以赴保增长，经济发展取得新成效。及时出台支持企业稳定生产经营的政策措施，全力抓好“六个一批”项目，加大力度扶持外经贸稳定发展，全市经济企稳回升。预计(下同)地区生产总值1473.84亿元，比上年增长(下称增长)11%；人均生产总值37158元，增长10.2%；地方公共财政预算收入总量突破百亿元，完成103.81亿元，增长12.6%；城镇居民人均可支配收入21754元，农村居民人均纯收入10615元，分别增长14.3%、17.8%。投资持续较快增长，固定资产投资852.6亿元，增长20.1%，144个重点项目完成投资计划；组织实施“六个一批”项目413宗，其中增资扩产和落地动工项目完成投资192.16亿元；贵广、南广铁路建设稳步推进，江肇高速二期建成通车，肇花高速、二广高速怀集至连州段和至贺州段建设进展顺利。外经外贸实现增长，合同利用外资29.1亿美元，实际吸收外资11.5亿美元，分别增长25.5%、11.9%，引进世界500强和境外大型企业投资项目4宗，第十届“肇庆金秋”引进投资489.2亿元；进出口总额63.5亿美元，增长11.2%，其中出口总额37.8亿美元，增长14.3%。第三产业平稳发展，社会消费品零售总额433.38亿元，增长15.1%；主要景区（点）接待人数2654.3万人次，旅游收入180.37亿元，分别增长10.4%、26.7%；港口货物吞吐量2747万吨，增长10%；商品房销售额175.82亿元，增长12.6%。

　　——加快推动新型工业化，产业转型升级迈出新步伐。肇庆高新区加快转型发展，大旺产业转移园实现扩园，亚洲铝业二期建成投产，国电热电联产等重大项目加快推进，引进恒迅蓝光盘等一批大项目，新增高新技术企业占全市三分之一，成为国家知识产权试点园区，与四会、高要、德庆、怀集共建产业园。工业经济快速发展，全市工业固定资产投资422.5亿元，增长33.8%，规模以上工业增加值674.63亿元，增长20.2%，六大支柱产业增加值增长23%。创新能力持续提升，实施重点技改项目55项，新建国家级创新平台2家，省级以上企业技术中心增至16家，高新技术企业增至91家，新增省著名商标20件，专利授权量增长38%，成为国家知识产权试点市。节能降耗成效明显，单位生产总值和工业增加值能耗分别下降3.6%、5%。

　　——加速推进新型城市化，宜居城乡建设实现新突破。出台加快推进新型城市化实施意见。肇庆新区规划建设全面启动并上升为省重大发展平台，总体规划获省政府审议通过，一批专项规划编制完成，“五个一”工程加快推进。肇庆市城市总体规划修编和“十二五”城镇化发展、创建宜居城乡等重点规划编制完成，中心城区基本实现控制性详细规划全覆盖，村庄规划覆盖率提高到56.6%。东调洪湖工程进展顺利，阅江大桥正式动工，西江路扩建改造、端州四路过街隧道等重点市政工程建成开通，续建和新增动工“三旧”改造项目88个，新建绿道169公里、城市绿道慢行道152公里。县（市）城市总体规划修编基本完成，新城区和中心镇、名镇规划建设加快推进。新创省卫生镇15个、省卫生村257个，新增省卫生村数量连续五年居全省首位。污染减排项目全面完成，大气、水体等环境质量稳定达标，城市绿化建设、环星湖绿道等项目获省宜居环境范例奖，高分通过国家环境保护模范城市复核验收。

　　——不断扩大深化合作，一体化进程步入新阶段。《珠三角规划纲要》“四年大发展”考核任务全面完成。《广佛肇经济圈发展规划》深入实施，39项重点合作项目进展顺利，广佛肇（怀集）经济合作区规划建设全面启动，广佛肇高速公路前期工作扎实推进，广佛肇城际轨道项目完成年度投资计划，水环境保护等环境联防联治成效初显，路桥通行费年票和移动通信资费实现一体化。与梧州市、贺州市签署战略合作框架协议。全年承接产业转移项目137宗（含意向），计划总投资368.62亿元。

　　——统筹推进区域经济，城乡发展呈现新亮点。完成市县两级基本农田保护示范区建设，通过省耕地保护责任考核，粮食连续6年增产，超额完成造林任务，实现农林牧渔业总产值370.43亿元，增长5.7%。中心区和山区生产总值均保持两位数增长，山区地方公共财政预算收入和规模以上工业增加值增速均高于中心区，农村居民人均纯收入增幅高于城镇居民人均可支配收入。各县(市、区)发展亮点纷呈。端州区华南智慧城成为省现代产业500强，中小企业服务中心被认定为省示范平台。鼎湖区公共财政预算收入、外贸出口等主要指标增速居全市前列，引进海印、美的等知名企业。四会市成为省可持续发展实验区，竞得省第四批水利建设示范县项目。高要市经济总量和财政收入稳居全市首位，大唐国际项目获省核准。广宁县华南再生资源产业园成为省市共建循环经济产业基地，引进境外大型企业维信集团。德庆县农村综合改革走在全市前列，成为全国农民林业专业合作示范县。封开县粤桂合作特别试验区上升为两广区域合作平台，华林化工中试基地等重点项目试投产。怀集县两广合作怀集八步试验区前期工作扎实推进，登云汽配通过上市审核。

　　——积极创新体制机制，重点领域改革创出新成绩。市直部门实现行政审批“两集中两到位”，企业登记相关领域审批制度改革全面推进，开通全国首创的行政审批“阳光通”短信服务系统，省网上办事大厅肇庆分厅基本建成。赋予高新区、新区一批市级管理权限，“两区”实行行政审批“零收费”。端州城区城市管理事权下放改革初见成效。县级大部制和简政强镇改革试点进展顺利。完成事业单位分类改革，累计减少事业单位476个。农村综合改革试点扎实推进，集体“三资”清理监管工作成效明显。市与端州区、高新区财政管理体制进一步理顺，“营改增”试点进展顺利，预算单位全部纳入国库集中统一支付，市本级公务卡使用改革全面完成。社会信用和市场监管两大体系建设加快推进，4个行业和1个综合地区试点有序开展。组建企业融资服务中心，新引进银行保险业机构7家。我市登记在册社会组织增至1138家，成为省社会创新实验基地，端州社区服务管理项目成为省社会创新试点。完成市级国有文艺院团转制，被评为全国文化体制改革先进地区。

　　——扎实办好民生实事，社会各项事业迈上新水平。加大民生投入，全市公共财政用于十一类民生支出119.25亿元，增长17.5%；投入6.97亿元全面完成市十件惠民实事。扶贫开发“双到”工作顺利完成。新增就业89117人，年末城镇登记失业率控制在2.6%以内。市场物价监管得到加强，新建平价商店92家，居民消费价格总指数涨幅控制在2.8%以内。城镇基本社会保险五大险种参保任务超额完成，城乡居民社会养老保险制度实现全覆盖，城镇居民医保和新型农村合作医疗实现制度整合。城镇、农村家庭年人均收入分别低于3480元、2760元的困难群众全部纳入低保，发放了老年人高龄津贴。建立救灾物资储备仓库和代储制度，创建全国综合减灾示范社区3个。累计投入26.3亿元，创建成为省教育强市；义务教育规范化学校覆盖率提高到84.2%，新增优质学前教育学位4000多个，建成广东信息工程学院。市第一人民医院新院和市中医院新住院大楼投入使用，社区卫生服务中心实现城市街道全覆盖，规范化卫生站实现行政村全覆盖，公办基层医疗机构全部实行基本药物零差率销售，免费提供11项基本公共医疗服务，开展四会市公立医院改革试点，农村中医工作走在全省前列。计生普惠政策全面落实，完成省下达人口控制计划。市图书馆新馆建成，公共文化服务网络更加完善，文化产业招商取得新成果。治安防控体系巩固提升，各类违法犯罪有效打击，“三打”专项行动成效明显，安全生产和食品药品安全形势平稳，社会大局和谐稳定。国防动员不断加强，双拥工作取得新成效。民政、民族、宗教、外事、侨务、港澳、体育、人防、统计、地方志、档案、气象、慈善、妇女儿童、残疾人等工作取得新成绩。各级驻肇单位为肇庆发展作出了积极贡献。

　　——切实加强自身建设，打造服务型政府取得新进展。认真学习贯彻党的十八大精神。坚持向市人大及其常委会报告工作，向市政协通报情况，自觉接受监督，按期办结人大代表建议97件和政协提案112件。制定实施依法行政工作定期报告、重大行政决策专家咨询论证等制度，认真听取各民主党派、工商联、无党派人士、各人民团体和专家顾问的意见建议。“六五”普法深入开展，被评为省首批法治城市创建先进单位。按时办理行政复议申请84件。政务公开深入推进。领导干部定期接访制度有效落实。党风廉政建设责任制更加健全，行政监察和审计监督不断加强，重点项目电子监察系统和公共资源电子监管平台建成使用。在全省率先开展“承诺、践诺、评诺”活动，“行风热线”、“网络问政平台”越办越好。

　　各位代表，去年取得的成绩来之不易。我代表市人民政府，向各位代表、全市人民和所有关心支持肇庆发展的各界人士表示衷心的感谢！我们也清醒认识到，我市发展还存在不少困难和问题：经济欠发达、发展不平衡仍未根本解决；受国内外复杂严峻的经济形势等因素影响，去年三项指标增幅未完成全年目标任务；科技创新能力不足，主导产业不够突出，现代服务业发展有待加快；基本公共服务均等化水平不高，社会建设创新力度有待加大，改善民生任务仍然艰巨；服务型政府建设存在薄弱环节，行政服务效能仍需进一步提升。我们必须采取更有力措施加以解决。

　　2013年工作安排

　　今年是全面贯彻落实党的十八大精神的开局之年。当前，世界经济复苏缓慢，国内经济企稳回升基础仍需巩固，总体经济形势仍然复杂多变。但是更要看到,党的十八大确立了全面建成小康社会的宏伟目标，习近平总书记寄望广东努力成为发展中国特色社会主义的排头兵、深化改革开放的先行地、探索科学发展的实验区，为我市提供更强大的发展动力。中央确定稳中求进的经济工作总基调，我省推动工业化、信息化、城镇化、农业现代化协调同步发展，为我市创造更有利的发展机遇。“两区”、“两化”发展提速，融入区域一体化步伐加快，绿色生态宜居环境优势凸显，为我市奠定更坚实的发展基础。我们必须坚定信心，把握机遇，应对挑战，真抓实干，迈出科学发展更好更快的新步伐。

　　今年政府工作的总体要求是：以邓小平理论、“三个代表”重要思想、科学发展观为指导，深入贯彻党的十八大、中央经济工作会议和习近平总书记视察广东重要讲话精神，全面落实省委十一届二次全会精神和省委胡春华书记对肇庆的重要指示，按照市委十一届三次全会决策部署，紧紧围绕主题主线，坚持把发展作为首要任务，大力实施“两区引领两化”战略，继续解放思想，深化改革开放，强化创新驱动，切实保持经济较快增长，切实推动产业转型升级，切实促进区域协调发展，切实加快宜居城乡建设，切实增进民生福祉，加快建设融入珠三角、连接大西南的枢纽门户城市，为尽快建设成为代表珠三角科学发展成果城市、与全省同步率先全面建成小康社会打下更加坚实基础。

　　今年我市经济社会发展主要预期目标是：生产总值增长11%，人均生产总值增长9.5%，规模以上工业增加值增长16.8%，固定资产投资增长18%，社会消费品零售总额增长12%，实际利用外资增长6%，外贸进出口总额增长5%，地方公共财政预算收入增长15%；居民消费价格总指数涨幅控制在3.5%以内；城镇居民人均可支配收入增长12%，农村居民人均纯收入增长13%；城镇登记失业率控制在2.6%以内；节能减排约束性指标完成省下达年度计划。为实现上述目标，要以深入实施“两区引领两化”战略为重点，着力抓好以下工作：

　　一、着力加快产业转型升级推动新型工业化。优化市域工业布局，推动各级各类园区主导产业集聚发展。加快推进高新区“二次创业”，推动大旺产业转移园“二次扩园”，实施高端产业集聚孵化工程，全力抓好2个以上投资超百亿元的重大产业项目动工建设。加大“一区多园”实施力度，推动高新区与各地分园优势互补、资源共享。加快推进产业结构调整，全力引进战略性新兴产业和先进制造业，改造提升传统产业，促进企业转型发展。重点围绕支柱产业引进行业龙头和重大项目，发展壮大优势产业集群。抓好工业项目促建、促产、促效，全力推进39项重大产业项目和135项重点增资扩产项目。加强企业融资服务，大力扶持中小微企业。落实改善创新环境五年行动计划，开展产业、科技、金融融合创新试点，推动区域创新和协同创新，培育壮大创新人才团队和创新型企业，组织实施一批产学研重大合作项目和高新技术产业化项目。加强工业设计研发推广。推进质量强市和标准化生产。

　　二、着力加快城市扩容提质推进新型城市化。坚持走集约、智能、绿色、低碳的新型城市化道路，落实加快城区扩容提质五年行动计划，编制实施市域城镇体系规划，推动新区引领、中心组团一体互动、山区腹地集约发展。完善肇庆市城市总体规划、肇庆新区总体规划和历史文化名城保护规划，加快编制实施各类专项规划和重点片区控制性详细规划。全力抓好肇庆新区建设发展，抓紧推进“五个一”工程，启动建设5个以上投资超30亿元的重点项目，加快筹建新区发展大厦、文化体育设施以及名校、名医院等重点工程，尽快建成新区基本框架。加强城东新区公共服务配套设施建设，大力推进北岭四区道路、东调洪湖、下湾湿地公园、鼎湖大道等市政工程，加快大冲交通枢纽、阅江大桥、桥北路立交建设，推进端州、鼎湖公共设施一体化，推动“一江两岸”对接发展。积极推进江滨西堤示范段等“三旧”改造项目。继续缓解城区交通拥堵问题。构建城市管理综合执法机制，继续推动城市管理重心下移，探索实施垃圾分类收集运输处理。加快各县（市）城区和中心镇总体规划修编，协调推进各县（市）旧城改造和新区开发，加快名镇名村建设，开展100条行政村村庄规划。积极推进“镇改街”、“村改居”，有序推进农业转移人口市民化。

　　三、着力落实《规划纲要》推动区域一体化发展。协同加快广佛肇经济圈建设，强化落实“五个一体化”专项规划，扎实推进重点合作项目，深化多领域合作。全力推动交通基础设施对接，积极推进贵广铁路、南广铁路、广佛肇城际轨道、广佛肇高速、肇花高速、二广高速怀集至连州段和至贺州段、321国道端州至鼎湖段升级改造等重点交通项目建设，扎实做好怀阳、汕湛高速前期工作，推动西江航道肇庆段创建全国文明样板航道，谋划柳肇铁路项目。全力打造区域合作平台，加快广佛肇（怀集）经济合作区、粤桂合作特别试验区、两广合作怀集八步试验区规划建设。推动“双转移”创新提质，实施“乡贤反哺”工程，优化承接项目结构，力促转移项目扩张升级发展。

　　四、着力加强招商选资促进重点项目建设。加快实施建设法治化国际化营商环境五年行动计划。创新招商引资方式，瞄准世界500强和国内外知名企业开展定向招商、产业链招商，引导外来投资投向先进制造业、高新技术产业、战略性新兴产业和现代服务业。着力提高招商项目履约率，增强招商实效。扎实推进“六个一批”项目，积极谋划一批重大项目进入国家、省计划，全力推动项目落地动工建设。协调推进鼎丰纸业二期、华润水泥第5-6线、封开铜钼矿、高新区国电、高要大唐、四会中电等重点产业项目。全年计划安排市重点项目100项，计划投资279.77亿元。

　　五、着力发展现代服务业提升第三产业水平。加快华南智慧城、中巴软件园等现代服务业集聚区建设。发展壮大肇庆新港、三榕港等物流基地，打造内河综合物流枢纽。抓紧实施培育幸福导向型产业体系五年行动计划。加快推进端砚文化特色产业集聚园、古宋城保护与开发等文化产业项目，打造一批文化产业示范基地。积极引进国际品牌酒店，全面提升星湖风景名胜区规划建设水平并力争创建成为5A景区，加强绿道网对接建设和管理，谋划建设大型生态休闲旅游产业园区，促进旅游业转型发展。加快创建外贸转型升级示范基地，扩大自主品牌和高附加值产品出口，重点支持再生资源和改善民生消费品进口，促进外贸稳定发展。积极发展电子商务，促进名优产品“网上行”，打造一批城市中心商业区和商贸物流龙头企业，做大做强端砚、玉器等专业市场，促进传统商贸业转型升级。推动房地产业平稳健康发展。拓展政银企合作平台，大力支持企业上市。

　　六、着力壮大县域经济统筹城乡协调发展。优化县域产业结构，推动德庆、怀集省级产业转移园扩园发展，推进高要创建省级汽车零部件产业专业园和广宁申办省级产业转移园，打造一批省级示范专业镇，大力承接主导产业项目，推动资源型工业延伸深加工链条。加快实施高标准基本农田工程和农田水利工程，抓紧推进西江干堤防洪系统工程。培育新型农业经营主体，做大做强现代农业园区，加强特色农产品专业市场建设，积极推广良种良法，大力发展绿色品牌农业。扩大珍贵树种种植规模，加快发展林产化工、林浆纸、板材加工等林产业，创建现代林业示范市。全面落实强农惠农富农政策，加快实施农村综合改革和征地制度改革，推进农村集体资产交易、土地承包经营权流转服务、集体“三资”监管等平台建设，创新农村社会管理服务。继续推进交通、电网、饮水安全等农村公用设施建设，统筹推进城乡教育、医疗、文化等公共资源均衡配置。组织开展新一轮扶贫开发。

　　七、着力深化重点领域改革增创体制新优势。抓好四会市行政体制综合改革先行先试试点，上半年完成各县（市）大部制改革，深化富县强镇和简政强镇事权改革。加快实施市县两级深化审批制度改革方案，实行行政审批事项目录管理制度和新设立行政审批事项公开听证制度，探索建立“宽入严管”的企业登记管理体制。推进行政审批全程网上电子化运转，抓紧完善省网上办事大厅肇庆分厅，加快市县镇三级联动行政服务体系建设并逐步向村、社区延伸。巩固扩大“三打”成果，扎实建设“两建”制度，健全竞争有序的市场环境。完善公共财政体系，加快建设公共资源交易平台，推进市属国有资产和行政事业单位经营性资产重组整合。实施社会管理信息化、培育发展社会组织、加强人口服务和管理、群团组织创新社会服务四大工程，扩大政府向社会组织购买服务，抓好端州社区服务管理试点。深化科技、文化等体制改革。

　　八、着力强化生态文明建设打造宜居美丽肇庆。出台实施市级主体功能区规划，优化市域产业区、生活区、生态区布局。加快实施创建幸福村居五年行动计划，广泛开展“美丽肇庆·幸福家园”创建活动，继续创建省卫生镇村和生态文明村，打造一批幸福安居示范村。抓好清洁生产，强化建筑节能，推广生活节能，完成公共照明领域普及LED照明任务，推进机动车污染物总量减排。强化环保准入和执法监督，加快推进中心镇污水处理“一镇一厂”建设，深化区域大气污染联防联控，抓好重金属等环保专项整治，着力解决热点环境问题。划定乡镇以上饮用水源保护区，加快实施水生态环境建设工程。加强自然生态和森林资源保护，实施生态景观林带、森林碳汇、森林进城围城三大生态工程。深入开展农村清洁工程，推进城乡生活垃圾无害化处理“一县一场、一镇一站、一村一点”建设。强化集约节约用地，构建城乡建设用地增减挂钩机制，遏制违法违规用地。加强地质灾害防治，加快乡镇防灾减灾网络全覆盖。

　　九、着力发展社会事业提升民生福祉。统筹实施基本公共服务均等化方案，让发展成果更多更公平惠及全市人民。完善公共就业服务体系，建立全员就业培训制度，支持青年创业，促进农村劳动力培训转移就业。加强劳动保障监察，积极创建和谐劳动关系示范区。加强社保扩面征缴，推进城乡养老保险衔接转换，探索建立大病保险制度和居民医保引入市场机制。把农村家庭年人均收入低于3000元的困难群众纳入低保，加强残疾人帮扶工作。优先发展教育，力争年内实现省教育强县全覆盖，启动创建教育现代化先进县(市、区)，深入实施学前教育三年行动计划，加大义务教育规范化学校建设力度，巩固提升“普九”、“普高”成果，支持肇庆学院“申硕改大”和肇庆科技学院、肇庆工商学院升格为本科院校，推动职教院校创建国家级示范学校。健全城乡医疗卫生服务体系，推进县级公立医院改革和基层医疗卫生机构综合改革，筹建市妇女儿童医疗保健中心，实施乡镇卫生院标准化建设，推动村级卫生站全部使用基本药物，加快中医药强市建设。深入创建全国文明城市。启动市博物馆、市文化馆、市青少年宫、市档案馆等重点文化场馆建设，繁荣群众文化，打造文艺精品，加强文化遗产保护。深入实施全民健身计划，积极申办第十五届省运会。加强人口计生综合治理，稳定低生育水平。做好第三次全国经济普查。推进人民武装、国防动员和双拥工作。充分发挥民政、民族、宗教、外事、侨务、港澳、人防、地方志、档案、气象、地震、慈善、妇女儿童、老龄等工作的重要作用。

　　十、着力加强政府自身建设提高法治服务水平。认真落实中央关于改进工作作风、密切联系群众八项规定，加快转变政府职能，努力构建职能科学、结构优化、廉洁高效、人民满意的法治服务型政府。自觉接受人大及其常委会法律监督和政协民主监督，定期向人大及其常委会报告工作和向政协通报情况，办好人大代表建议和政协提案。认真听取各民主党派、工商联、无党派人士、人民团体、专家顾问意见建议，落实重大行政决策专家咨询论证制度。继续推进“六五”普法，开展法治市、县创建活动。加强政务诚信建设，健全政务公开和各领域办事公开制度，实施“阳光复议工程”。完善立体化社会治安防控体系，加强公安信息化建设，依法防范和惩治违法犯罪。提高应急管理能力。狠抓安全生产、食品药品安全、产品质量、市场物价等民生热点领域监督执法。健全政务督查和行政效能监察。强化落实领导干部定期接访和下访约访制度，办好“行风热线”、“网络问政”等平台，及时解决群众关心的热点难点问题。强化党风廉政建设责任制，扎实推进惩防腐败体系建设，加强审计监督，加大纠风力度，推进“三公”经费公开和公车改革。

　　各位代表，在落实今年省十件民生实事有关任务的基础上，市政府将继续办好十件惠民实事：一是推进就业创业。支持2600人创业，新增城镇就业46000人，为2600名异地务工人员实行减免费技能培训。二是促进教育均衡。创建8个省教育强镇，广宁、怀集县创建成为省教育强县。中等职业（技工）教育免学费政策实施范围扩大至所有农村（含县镇）户籍全日制在校生。三是推动稳价惠民。继续向全市10.8万名低收入群体和困难群众发放一次性临时价格补贴。推进3个蔬菜基地建设。新建平价商店30家。端州区创建4个以上、其他县（市、区）和高新区分别创建1个以上标准化惠民农贸市场。四是加强医疗服务。市、县级公立医院使用基本药物比例分别达30%和40%以上，创建1家平价医院、21个平价诊室，所有政府办基层医疗机构推广平价药包。城乡居民医疗保险覆盖率达到98%以上。医保经办服务网点覆盖到全市所有乡镇（街道）。全面实施免费孕前优生健康检查。五是健全食品药品监测网络。建成市食品药品检验服务中心，两年内形成覆盖各县(市、区)并逐步延伸到乡镇的监测网络。六是加强住房保障。全市新增保障性住房2950套（户）。全面启动709户水上居民安居工程。七是改造城乡道路。动工建设广佛肇高速公路。完成农村公路硬底化300公里。端州区、鼎湖区分别改造10条和5条旧街旧巷。八是推进养老助困。城镇和农村低保平均补差水平分别提高到每人每月242元、125元，农村五保供养标准不低于当地上年农村居民人均纯收入的60%。端州区建设2个、其他县（市、区）和高新区分别建设1个居家养老服务中心示范点。九是优化文体服务。新建和改扩建（或完善设施设备）乡镇（街道）综合文化站20个、行政村（社区）文化室公共电子阅览室100个。端州区创建5个、其他县（市、区）分别创建1个广场文化体育活动示范点。建设乡镇农民体育健身工程项目10个。放映农村公益电影16000场。全面免费开放基层公共文化设施。十是加强生态环境建设。四会市、广宁县、封开县和怀集县建成生活垃圾无害化填埋场，全市建制镇均建成1座生活垃圾转运站，自然村均建成1座以上生活垃圾收集点。继续推进星湖水质整治工程。新增投放新能源公交车50台。建设生态景观林带14.8万亩、碳汇工程林6万亩。各县(市、区)创建市级以上生态村（镇）不少于2个。

　　各位代表！肇庆人民对美好生活的向往就是我们的奋斗目标。让我们在中共肇庆市委的正确领导下，深入贯彻党的十八大精神，团结依靠全市人民，解放思想，改革创新，脚踏实地，真抓实干，为把肇庆建设成为代表珠三角科学发展成果城市、全面建成小康社会而努力奋斗！
